# Measuring Software Maintainability with Naïve Bayes Classifier

**DOI:** 10.3390/e23020136

**Published:** 2021-01-22

**Authors:** Nayyar Iqbal, Jun Sang, Jing Chen, Xiaofeng Xia

**Affiliations:** School of Big Data & Software Engineering, Chongqing University, Chongqing 401331, China; nayyariqbal@cqu.edu.cn (N.I.); jingchen@cqu.edu.cn (J.C.); xiaxiaofeng@cqu.edu.cn (X.X.)

**Keywords:** errors, Naïve Bayes, software components, software requirements, supervised learning, WEKA software

## Abstract

Software products in the market are changing due to changes in business processes, technology, or new requirements from the customers. Maintainability of legacy systems has always been an inspiring task for the software companies. In order to determine whether the software requires maintainability by reverse engineering or by forward engineering approach, a system assessment was done from diverse perspectives: quality, business value, type of errors, etc. In this research, the changes required in the existing software components of the legacy system were identified using a supervised learning approach. New interfaces for the software components were redesigned according to the new requirements and/or type of errors. Software maintainability was measured by applying a machine learning technique, i.e., Naïve Bayes classifier. The dataset was designed based on the observations such as component state, successful or error type in the component, line of code of error that exists in the component, component business value, and changes required for the component or not. The results generated by the Waikato Environment for Knowledge Analysis (WEKA) software confirm the effectiveness of the introduced methodology with an accuracy of 97.18%.

## 1. Introduction

Software engineering consists of process, product, and resources entities. Processes are related to software activities, e.g., specification, design, or testing [1]. Products are correlated to documents which result from the activity of the process, e.g., specification, design document, deliverables, artifacts, etc. [2]. Resources are related to entities that are considered by the process activity, e.g., personnel, CASE tools, or hardware [3]. The entities consist of external and internal attributes. External attributes describe the entity behavior while internal attributes are those that portray the entity itself. In literature [4], machine learning applications are incorporated with software engineering tasks for: (i) predicting external or internal attributes of resources, product, or process, (ii) recycling of processes or product, (iii) improving the processes by retrieving the specification, etc.

In this research, we measure software maintainability with a Naïve Bayes classifier. This research also classifies the software components that do not require modification, based on whether it contains errors or not. Low quality requirements [5] and software components can provoke errors or defects in the software, which increases the software development cost. The requirements engineering process and design of software components are considered an important factor in software development.

Requirements engineering is defined as a “systematic process of developing requirements through an iterative co-operative process of analyzing the problem, documenting the resulting observations in a variety of representation formats, and checking the accuracy of the understanding gained” [6]. Component are defined by Scott and Morgado [7] “as an independent piece of software. This standalone, discrete piece of software has a clear boundary that is accessible via an API and contains all of the application dependencies. This enables teams to build the user interface quickly, leveraging the library of components”, e.g., freeze-user, close, and download are represented by 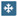, 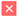, and 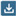, user interfaces, respectively. 

Software maintainability can be measured either in reverse or forward engineering. In this research, authors measure a quality attribute (e.g., maintainability). According to IEEE standard glossary of software engineering terminology, software maintainability is defined as “the ease with which a software system or component can be modified” [8]. The maintainability process starts after the delivery of software, which is the key stage of the system development life cycle. Software requires maintainability to remove faults, improve performance, or to adopt the software according to modified environment [9]. Due to COVID-19 (coronavirus disease 2019), business organizations brought revolutionary changes in their working environment and education institutes changed to e-learning. The objective of this research is to measure the software maintainability (e.g., by Naïve Bayes classifier) and to extract the errors from the software components.

Supervised learning is that in which we have input data and an expected output result. Supervised learning is grouped into classification and regression problems [10]. If any type of change is required or the software component does not provide the required functionality according to user new requirements, then type of error or defect is reported else it is a correct component. Types of errors or defects are described in detail in Section 1.1. The supervised learning approach is applied to extract errors in the software components. Details on how to extract errors by supervised learning are discussed in Section 3. 

Each software component is validated against its specification. Software components are represented by a component-based user interface. In this research, we validated the software requirements using requirements a validation framework [11]. Machine learning techniques such as Naïve Bayes classifier are applied for measuring software maintainability and also determine the business value of software. The business value of the software determines whether the software requires maintainability by reverse engineering or by forward engineering. If the business value of software is high, then software is maintained by reverse engineering, else by forward engineering [12].

The designed dataset consisted of following attributes: component-state, successful/error-type, error-LOC, business-value and changes. The component-state attribute determines whether the software components require small-changes, average-changes, or superior-changes, or is accurate-component. The successful/error-type attribute specifies the type of error in the software component or it is the correct component. The error-LOC attribute represents the number of lines of the code (LOC) that had error in it. The business-value attribute specifies whether the software component has business value or not (e.g., true or false). The changes attribute determines whether the software component requires changes or not (e.g., yes or no). Values were assigned to the attributes of the dataset with the collaboration of software engineers of the software company. The introduce approach helps to improve the performance of software, remove faults, and extract the errors from exact software component, and by this approach software is easily modified. This approach also keeps a record regarding the software maintainability in the dataset.

### 1.1. Types of Errors/Defects

The quality problems that are identified in the software before it is handed over to end-users are called errors whereas if they are identified after the software has been handed over to end-users are called defects. In this research, authors monitored the following types of errors in the software [12].

#### 1.1.1. Incomplete Erroneous Specifications (IES)

Incomplete or erroneous specification (IES) result from deviations from manual process, lacking or partial implementation of software specification. IES occurs if goals and objectives of software are not completely assembled in the functional and non-functional requirements [13].

#### 1.1.2. Misinterpretation of Customer Communication (MCC)

Misinterpretation of customer communication (MCC) errors occur due to incorrect extraction of requirements from user stories during requirements gathering phase or due to negligence of not adopting the requirement gathering techniques in the software development [12]. For example, consider the software component that was developed due to MCC from the requirement $R_{n}$. In this software component, online fee payment was only by credit card. During the validation process, a MCC error was found in the software component. Therefore, $R_{n}$ was corrected to $R_{n1}$ and $R_{n2}$. The following payment options were made to be available in the software component: credit card, PayPal, Apple Pay, Alipay, Western Union, Union Pay International. Rejected requirement “$R_{n}$: The user will be able to pay online fee by credit card”. Corrected requirement “$R_{n1}:$ The system shall display online payment options, $R_{n2}:$ The user shall be able to select one online payment option from the system”.

#### 1.1.3. Intentional Deviation from Specification (IDS)

Intentional deviation from specifications (IDS) result from negligence of the software engineers. In this, basic approved requirements are missed during the implementation of software components without any appropriate reason. Often, software developed from component-based development requires removal of extra functionalities or alteration in the software components in order to satisfy the approved requirements [14]. Consider the requirement number *m* of the software. “$R_{m}:$ The administrator of the company will be able to calculate the employees over time charges according to company rules and regulations”. According to the rules and regulations of the company if any employee is on half day leave or short leave in any working day and if the leave employee works in the evening time in the same day for over time charges, then the number of overtime hours of that employee will be included in the number of leave time hours. After this, the hours that are more than the morning working hours will be paid. For full day leave, the employee of the company will not be eligible to work in the evening time for same day. In the developed software, there was an error of IDS in which the software was calculating the overtime charges by ignoring the rules and regulations of the leave.

#### 1.1.4. Violation of Programming Standards (VPS)

When any modification is done in the programming standards by the software engineers or it is deviation from the programming standards then this injects violation of programming standards (VPS) errors in the software [15].

#### 1.1.5. Error in Data Representation (EDR)

Data formats must be specified in the software specification or software architecture, any negligence of this results in error in data representation (EDR) [16]. In order to avoid EDR, it is recommended to use data modeling tools such as [17] Visio, StarUML, Erwin, Entity Framework Add-on, DataArchitect, ConceptDraw, CASEWise, CA Gen, Altova Database Spy, etc.

#### 1.1.6. Error in Design Logic (EDL)

Software design can be illustrated by Unified Modeling Language (UML) [18], Data flow Diagram (DFD) [19], or Entity Relationship Diagram (ERD) [20]. If there is any error in the software component due to error in design, then this type of error is called error in design logic (EDL). Causes of error include elimination of vital system states and elimination of procedures that were responsible for reporting prohibited operations.

#### 1.1.7. Inconsistent Component Interface (ICI)

**Figure 1:**
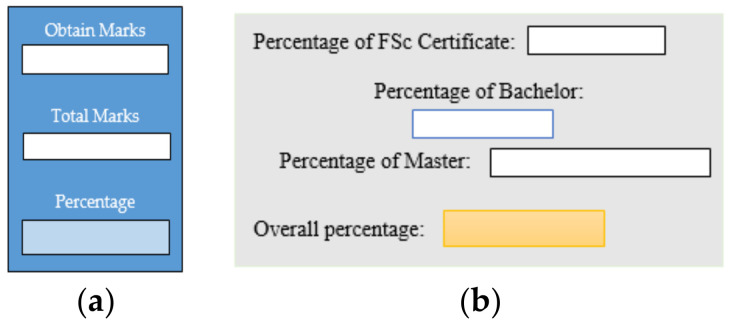
Component interfaces: (**a**) recommended component interface, (**b**) inconsistent component interface.

Inconsistent component interface (ICI) errors are result from violation in the recommended visuals designs, layouts, and standards [21]. Consider Figure 1 that represents the component interfaces.

#### 1.1.8. Incomplete or Erroneous Testing (IET)

The software that is developed according to approved requirements and by using modeling tools still contains errors or defects because during the testing phase, verification of code was not properly performed. These errors or defects result when some modules of the software are not tested, testing is not done thoroughly, failure to correct reported faults due to limitation of time, or incorrect identification of error location [22].

#### 1.1.9. Inaccurate or Incomplete Documentation (IID)

The incomplete user manuals in the documentation often operates the software with erroneous results. If the documentation does not support implementation design, in future any modification in the software leads to software failure [23].

### 1.2. Disruptive Change

Heraclitas was a Greek philosopher famous for his opinion that constant change is the basic reality of this universe. This reality has been observed in every part of the history, as quickly evolving societies have regularly faced eras of discovery, disruptive change, and innovation. Gradual change occurs step by step and allows societies to adjust in it accordingly, whereas disruptive change is a dominant force that blasts on the scene by presenting unseen practices and new solutions [24]. 

**Figure 2:**
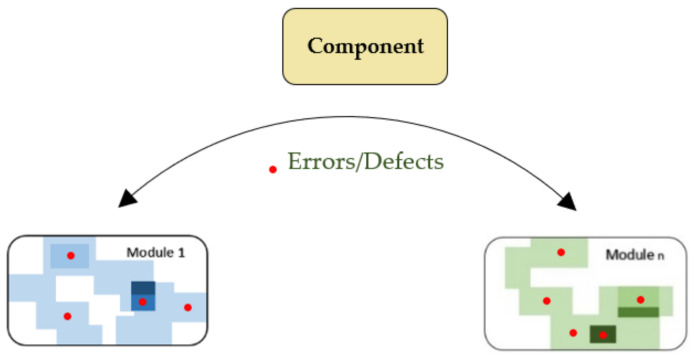
Errors or defects due to disruptive change.

In disruptive change, errors or defects occurs in the software due to unexpected movement of components from one module to another module. These changes can be in software specification or design of software and/or modules of software. Figure 2 represents the errors or defects that occur due to disruptive change.

## 2. Related Work

**Figure 3:**
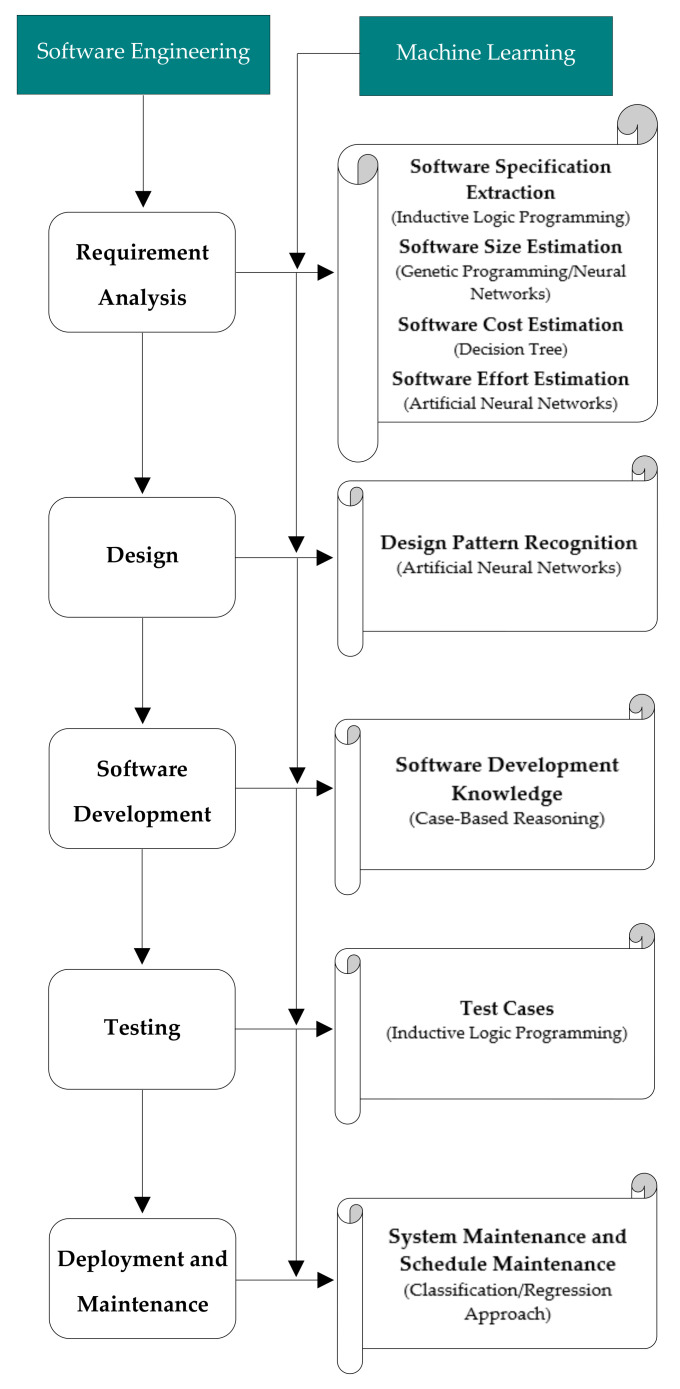
Extracting software engineering activities by machine learning techniques.

In this section, we discuss how researchers have used machine learning techniques in their research to solve problems related to software engineering. In [25], scenario based requirements Engineering (RE) was supported by concept learning (CL) for extracting requirements and goals from system specification. In [26], researchers used genetic programming for generating software quality models. Software metrics were collected before software implementation, then these were entered as input. During testing or deployment, genetic programming predicted the quantity of faults for each module. Figure 3 illustrates the extracting of software engineering (SE) activities at different phases of system development life cycle (SDLC) by machine learning (ML) techniques [4].

In [27], Cohen used inductive logic programming (ILP) for extracting functional and non-functional requirements from the software. In this paper [28], researchers estimated line of code from the function point of software by applying neural network (NN) and genetic programming (GP) algorithms of machine learning. In [29], genetic programming (GP) and a neural network (NN) were used to calculate software size in line of code (LOC) by validating the component-based methods. Briand et al. in [30] described a technique named optimized set reduction which is based on decision tree (DT) learning. This technique is used to estimate software cost by examining data of software engineering. In the study [31] conducted by Ertugrul et al., several algorithms of machine learning were examined with feature transformation, feature selection, and also with the techniques of parameter optimization. They introduced a new model which provides improved effort estimation by considering the artificial neural networks (ANN) specifically “multilayer perceptron topology”.

Alhusain et al. in [32] proposed approach for design pattern recognition which is based on machine learning technique called artificial neural network (ANN). For each design pattern or role, a separate ANN was trained with feature vector as diverse input. In [33], tools were introduced for the management of knowledge regarding software development through case-based reasoning. In [34], researchers discussed how to generate test cases for the testing of software based on inductive logic programming (ILP). Machine learning techniques [35] such as classification approach and/or regression approach can be used for the prediction of system maintenance in order to monitor the system from future failures and schedule in advance the system maintenance. 

In literature, different machine learning techniques have been used to extract different software engineering activities. In addition, to the best of the authors’ knowledge no related work or guideline has been found in which software maintainability has been measured by the authors’ introduced approach. In order to measure software maintainability in accurate form, the authors analyzed the software manually, e.g., identification of component state, errors in LOC per component, business value of component, and change required in a component or not. Error types were identified by supervised learning. Based on these five attributes, software maintainability was measured. Software size depends upon number of software components.

## 3. Material and Methods 

**Figure 4:**
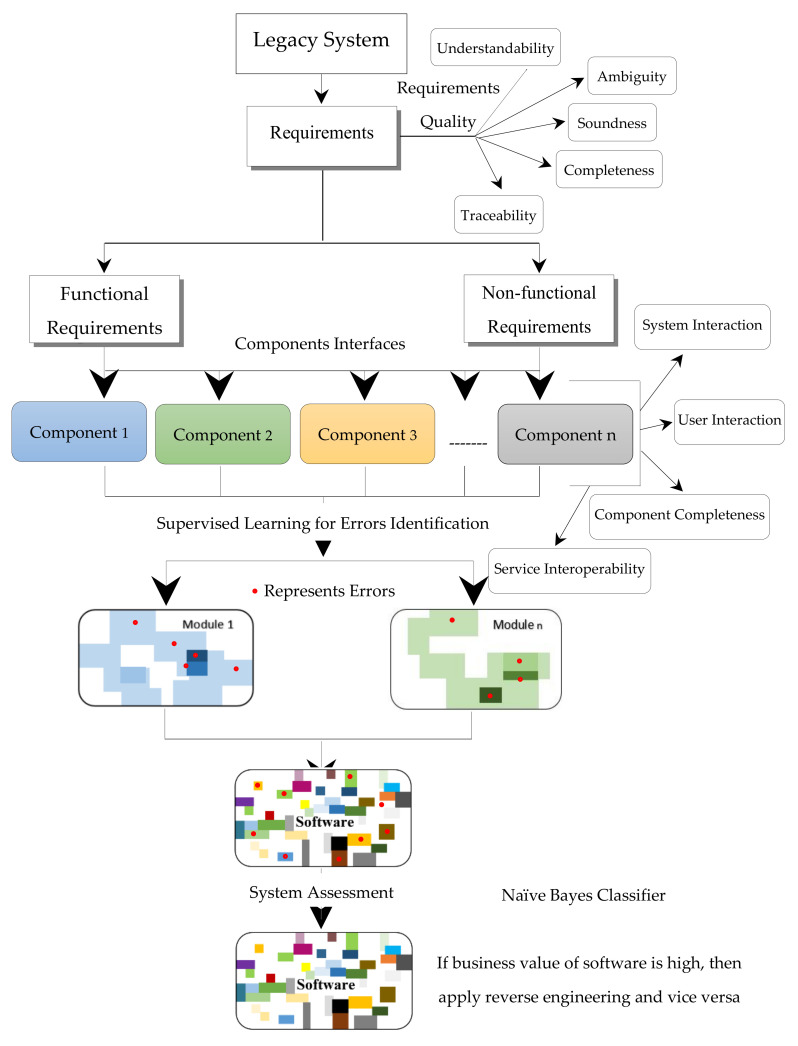
Software maintainability process

In this research, a case study was conducted in order to determine the business value of the software. The business value of the software determines which approach is feasible for software modification forward engineering or reverse engineering. Figure 4 represents the software maintainability process. The red circle represents the errors. The white portion in module/software represents a component with single function whereas colored portion represents components with multiple functionalities. The increase in shade of any color indicates the increase in functionalities of the component. In this research, software specification of the new software was checked against its recently developed components/modified components of legacy system in order to identify the type of errors that exists in it.

Let B and C represents the module 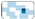 and component 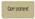 of the executable code of software (e) 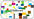.
e: B → C 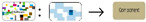


The maintenance of the legacy system was accomplished by component-based development. Therefore, each functionality of the software was monitored with the new demanded functional and nonfunctional requirements during the validation process. Each component, module, and the complete software was examined with respect to IES, MCC, IDS, VPS, EDR, EDL, ICI, IET, and IID. Let F represents the specification of the e.

e: C → F

In this research, software defects were detected by supervised learning. In this input, data were entered and the output results were matched with the expected output demanded by the user of software. If there was any contradiction between the system output and the expected output, then type error was identified. As defined by Murphy [10],

(1) $$T\;=\;{\left\{[x_{i},\;y_{i}]\right\}}_{i=1}^{n}$$

T stands for training set, whereas input is represented by x and output by y. Based on the test cases designed $x_{i}$, $y_{i}$ valued were entered, the type of errors that were identified are represented in Table 1.

Table 1 represents the validation of the legacy system by supervised learning approach, in which software components are represented by component-based user interfaces. New software components ($SC_{72}$ to $SC_{88}$) are represented in module 9 ($M_{9}$) in Table 1. Modules, software components, requirements, system response, successful, error type are denoted by upper case letters M _(1 ≤ *i* ≤ n)_, SC _(1 ≤ *j* ≤ n)_, R _(1 ≤ *l* ≤ n)_, SyRp _(1 ≤ *m* ≤ n)_, Sf, Et respectively whereas lower case letters such as *i*, *j*, *l*, *m* denote the range of indexes. $M_{i}SC_{j}$ stands for *j*th software component of *i*th module. In Table 1, requirements are written in standard format as “The system shall…”. In this research, only errors were monitored, if the software component is error-free then it is called correct component represented by CC (successful). If there is any error, then type of error is mentioned; type of errors are as described in Section 1.1. The asterisk * in red indicates that it is mandatory for the user to enter the data. When client identified what the system must not consist of, then these requirements are called inverse requirements. Inverse requirements can be functional and/or non-functional [36].

In $SC_{4}$, there was an error of IDS because in this, an alternative email address was accepted in the text box, whereas the text box was to confirm the email address that was entered in $SC_{3}$. Suppose user email address is abcde@hotmail.com. System response and expected system response are represented by $SyRp_{j.m}$ and $ESyRp_{j.k}$ respectively. *j*, *m*, and *k* denote the indexes of software component (1 ≤ *j* ≤ n), system response (1 ≤ *m* ≤ n) and expected system response, (1 ≤ *k* ≤ n). 

${\mathrm{Input}}_{1}$: if email address: abcde@hotmail.com → $SyRp_{4.1}$: email address verified

$ESyRp_{4.1}$: email address verified 

${\mathrm{Input}}_{2}$: if email address: jklmno@hotmail.com → $SyRp_{4.2}$: email address verified (Error type: IDS) 

$ESyRp_{4.2}$: email address does not match the confirm email address

Output: Error type: IDS

Expected output: CC

In $SC_{5}$, there was an error of IET because the system was accepting the application of applicants above 35 years. Inverse requirement: the system does not accept the application if the age of applicant is more than 35 years before the deadline of application. The text box will become red in color indicating that applicant is not eligible. Cell number consists of eleven digits.

${\mathrm{Input}}_{1}$: if date of birth: 1990/01/01 → $SyRp_{5.1}$: system stores the date of birth in the record 

$ESyRp_{5.1}$: system stores the date of birth in the record

${\mathrm{Input}}_{2}$: if date of birth: 1984/01/01 → $SyRp_{5.2}$: system stores the date of birth in the record (Error type: IET)

$ESyRp_{5.2}$: applicant is not eligible to apply

Output: Error type: IET

Expected output: CC

**Figure 5:**
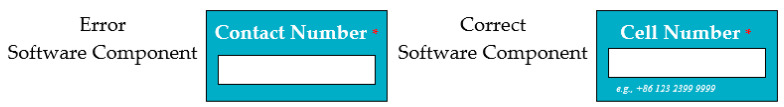
Software components.

In $SC_{6}$, there was an error of MCC because the system was also generating a PIN for landline telephones. Inverse requirement: the system will not send a PIN code to a landline telephone. Figure 5 represents the software components, error (MCC) and the correct software component. 

${\mathrm{Input}}_{1}$: if cell number: +86-123-2399-9999 → $SyRp_{6.1}$: cell number is updated in the system

$ESyRp_{6.1}$: cell number is updated in the system 

${\mathrm{Input}}_{2}$: if cell number: 123-7777-2222 → $SyRp_{6.2}$: please enter the country code 

$ESyRp_{6.2}$: please enter the country code

${\mathrm{Input}}_{3}$: if cell number: +86-123-4444 → $SyRp_{6.3}$: cell number is updated in the system (Error type: MCC) 

$ESyRp_{6.3}$: please enter cell number in correct format

Output: Error type: MCC

Expected output: CC

In $SC_{17}$, there was an error of EDR, because the date was wrongly formatted in the implementation, i.e., YYYY/DD/MM whereas the correct format was YYYY/MM/DD. Inverse requirement: expiry date of passport must be more than 6 months before the deadline of application. If the expiry date of passport is less than 6 months, then the system shall display error message (displays the text box for expiry date in red color) and asks the user to reenter the renew passport details.

${\mathrm{Input}}_{1}$: if passport number: AB123456 → $SyRp_{17.1}$: system stores the passport number in the record 

$ESyRp_{17.1}$: system stores the passport number in the record

${\mathrm{Input}}_{2}$: if issue date: 2017/12/31 → $SyRp_{17.2}$: system stores the passport issue date in the record $ESyRp_{17.2}$: system stores the passport issue date in the record

${\mathrm{Input}}_{3}$: if issue date: 2017/31/12 → $SyRp_{17.3}$: system stores the passport issue date in the record (Error type: EDR)

$ESyRp_{17.3}$: please enter issue date according to the format YYYY/MM/DD

${\mathrm{Input}}_{4}$: if expiry date: 2022/12/30 → $SyRp_{17.4}$: system stores the passport expiry date in the record

$ESyRp_{17.4}$: system stores the passport expiry date in the record

${\mathrm{Input}}_{5}$: if expiry date: 2020/30/12 → $SyRp_{17.5}$: system stores the passport expiry date in the record (Error type: EDR)

$ESyRp_{17.5}$: please enter expiry date according to the format YYYY/MM/DD

${\mathrm{Input}}_{6}$: if expiry date: 2021/03/15 → $SyRp_{17.6}:$ please enter the renew passport details

$ESyRp_{17.6}$: please enter renew passport details

Output: Error type: EDR

Expected output: CC

In $SC_{26}$, there was an error of IET because the system was also accepting the date with validity of three months. Inverse requirement: if the validity of address is less than 6 months, the system shall display error message to reenter the address with more than 6 months of valid date.

${\mathrm{Input}}_{1}$: if address is valid until: 2025/01/15 → $SyRp_{26.1}$: address date is updated in the system

$ESyRp_{26.1}$: address date is updated in the system

${\mathrm{Input}}_{2}$: if address is valid until: 2021/01/15 → $SyRp_{26.2}$: address date is updated in the system (Error type: IET)

$ESyRp_{26.2}$: please reenter the address with more than 6 months’ validity date

Output: Error type: IET

Expected output: CC

In $SC_{27}$, there was an error of EDL because the system was not sending the email to the relevant professors to whom the applicant research interests matches with, when the application was submitted.

${\mathrm{Input}}_{1}$: the applicant selects the research interests from the drop-down list → $SyRp_{27.1}$: the system sent the email to all the professors (Error type: EDL)

$ESyRp_{27.1}$: the system sent the email to the relevant professors to whom the applicant research interests match with

Output: Error type: EDL

Expected output: CC

In $SC_{49}$, there was an error of IES because the update option was providing the delete functionality.

${\mathrm{Input}}_{1}$: if user clicks on update option to update the entered information such as ISSN, authors name, article title, journal title, volume number, issue number, pages, year → $SyRp_{49.1}$: the required information has been deleted (Error type: IES)

$ESyRp_{49.1}$: please click enter the information

Output: Error type: IES

Expected output: CC

In $SC_{51}$, there was an error of VPS because when conference was selected in publication category software components (from $SC_{41}$ to $SC_{48}$) of journal were displayed instead of conference software components. 

${\mathrm{Input}}_{1}$: user selects the publication category e.g., conference → $SyRp_{51.1}$: the system displays software components from $SC_{41}$ to $SC_{48}$ e.g., ISSN, author names, article title, journal title, volume number, issue number, pages, year (Error type: VPS)

$ESyRp_{51.1}$: the system displays software components from $SC_{52}$ to $SC_{58}$ e.g., ISSN, authors name, article title, conference name, location (city, country), date, pages

Output: Error type: VPS

Expected output: CC

In $SC_{60}$, there was an error of EDR because the entered details of the conference article were displayed in journal reference format.

${\mathrm{Input}}_{1}$: user clicks on the update option → $SyRp_{60.1}$: the system displays the entered details according to journal reference format (Error type: EDR)

$ESyRp_{60.1}$: the system displays the entered details according to conference reference format

Output: Error type: EDR

Expected output: CC

$SC_{72}$: ${\mathrm{Input}}_{1}$: if student ID: *MSSE201701* → $SyRp_{72.1}$: student ID verified

$ESyRp_{72.1}$: student ID verified 

${\mathrm{Input}}_{2}$: if student ID: *SE201803* → $SyRp_{72.2}$: this is not valid student ID

$ESyRp_{72.2}$: this is not valid student ID

Output: CC

Expected output: CC

$SC_{73}$: ${\mathrm{Input}}_{1}$: if student full name: Rachel Melo → $SyRp_{73.1}$: student name verified

$ESyRp_{73.1}$: student name verified

${\mathrm{Input}}_{2}$: if student full name: Glaucia Vital → $SyRp_{73.2}$: this is not registered name in the system

$ESyRp_{73.2}$: this is not registered name in the system

Output: CC

Expected output: CC

$SC_{74}$: ${\mathrm{Input}}_{1}$: if passport number: ABC123456 → $SyRp_{74.1}$: passport number saved in the system $ESyRp_{74.1}$: passport number saved in the system

Output: CC

Expected output: CC

$SC_{75}$: ${\mathrm{Input}}_{1}$: if user selects PhD as his/her student category → $SyRp_{75.1}$: system updates PhD as his/her student category in the system

$ESyRp_{75.1}$: system updates PhD as his/her student category in the system

${\mathrm{Input}}_{2}$: if user selects Master as his/her student category → $SyRp_{75.2}$: system updates Master as his/her student category in the system

$ESyRp_{75.2}$: the system updates Master as his/her student category in the system

${\mathrm{Input}}_{3}$: if user selects Bachelor as his/her student category → $SyRp_{75.3}$: system updates Bachelor as his/her student category in the system

$ESyRp_{75.3}$: the system updates Bachelor as his/her student category in the system

Output: CC

Expected output: CC

$SC_{76}$: ${\mathrm{Input}}_{1}$: student selects his/her address from the available options → $SyRp_{76.1}$: system updates the address of the student in the system

$ESyRp_{76.1}$: system updates the address of the student in the system

Output: CC

Expected output: CC

$SC_{77}$: ${\mathrm{Input}}_{1}$: student enters the name and contact number in emergency details → $SyRp_{77.1}$: system saves the name and contact number in the emergency details

$ESyRp_{77.1}$: system saves the name and contact number in the emergency details

Output: CC

Expected output: CC

$SC_{78}$: ${\mathrm{Input}}_{1}$: if first entry date: 2019/09/31 → $SyRp_{78.1}$: first entry date is updated in the system

$ESyRp_{78.1}$: first entry date is updated in the system

${\mathrm{Input}}_{2}$: if first entry date: 2019/31/09 → $SyRp_{78.2}$: please enter date in correct format (YYYY/MM/DD)

$ESyRp_{78.2}$: please enter date in correct format (YYYY/MM/DD)

${\mathrm{Input}}_{3}$: airport city: Chongqing → $SyRp_{78.3}$: airport city name updated in the system

$ESyRp_{78.3}$: airport city name updated in the system

Output: CC

Expected output: CC

$SC_{79}$: ${\mathrm{Input}}_{1}$: user selects one option regarding COVID-19 test (negative, positive, not tested) → $SyRp_{79.1}$: system saves the results of COVID-19 test

$ESyRp_{79.1}$: system saves the results of COVID-19 test

Output: CC

Expected output: CC

$SC_{80}$: ${\mathrm{Input}}_{1}$: user enters the visa number → $SyRp_{80.1}$: visa number is updated in the system

$ESyRp_{80.1}$: visa number updated in the system

${\mathrm{Input}}_{2}$: if visa expiry date: 2021/12/31 → $SyRp_{80.2}$: visa expiry date is updated in the system

$ESyRp_{80.2}$: visa expiry date is updated in the system

${\mathrm{Input}}_{3}$: if visa expiry date 2021/31/12 → $SyRp_{80.3}$: please enter date in correct format (YYYY/MM/DD)

$ESyRp_{80.3}$: please enter date in correct format (YYYY/MM/DD)

Output: CC

Expected output: CC

$SC_{81}$: ${\mathrm{Input}}_{1}$: student enters his/her student ID: MSSE201701 → $SyRp_{81.1}$: student ID is verified

$ESyRp_{81.1}$: student ID is verified

${\mathrm{Input}}_{2}$: student enters the end date of degree: 2021/12/31→ $SyRp_{81.2}$: end date of degree is verified

$ESyRp_{81.2}$: end date of degree is verified

Output: CC

Expected output: CC

$SC_{82}$: ${\mathrm{Input}}_{1}$: if user enters ISSN: 1234–5678 → $SyRp_{82.1}$: system displays that the journal is recognized by SCI, web of science, and CCF

$ESyRp_{82.1}$: system displays that the journal is recognized by SCI, web of science and CCF

${\mathrm{Input}}_{2}$: if user enters title SEDB → $SyRp_{82.2}$: system displays that the conference is recognized by EI, and CCF 

$ESyRp_{82.2}$: system displays that the conference is recognized by EI and CCF

Output: CC

Expected output: CC

$SC_{83}$: ${\mathrm{Input}}_{1}$: if user enters the number of published articles in the indexing e.g., SCI, ESCI, CCF, EI → $SyRp_{83.1}$: system displays candidate is eligible

$ESyRp_{83.1}$: system displays candidate is eligible 

${\mathrm{Input}}_{2}$: if user does not enter any number of published articles in the indexing e.g., SCI, ESCI, CCF, EI → $SyRp_{83.2}$: system displays candidate is not eligible 

$ESyRp_{83.2}$: system displays candidate is not eligible

Output: CC

Expected output: CC

$SC_{84}$: ${\mathrm{Input}}_{1}$: if final defense date: 2021/12/31 → $SyRp_{84.1}$: final defense date is updated in the system

$ESyRp_{84.1}$: final defense date is updated in the system

${\mathrm{Input}}_{2}$: if final defense date: 2021/31/12 → $SyRp_{84.2}:$ please enter date in correct format (YYYY/MM/DD)

$ESyRp_{84.2}$: please enter date in correct format (YYYY/MM/DD)

Output: CC

Expected output: CC

$SC_{85}$: ${\mathrm{Input}}_{1}$: user enters student ID and supervisor name → $SyRp_{85.1}$: system displays defense information (student ID, thesis title, date, time, location)

$ESyRp_{85.1}$: system displays defense information (student ID, thesis title, date, time, location)

Output: CC

Expected output: CC

$SC_{86}$: ${\mathrm{Input}}_{1}$: user enters student ID and school name → $SyRp_{86.1}$: system generates transcript

$ESyRp_{86.1}$: system generates transcript

Output: CC

Expected output: CC

$SC_{87}$: ${\mathrm{Input}}_{1}$: user enters student ID and degree completion date → $SyRp_{87.1}$: system generates university clearance form

$ESyRp_{87.1}$: system generates university clearance form

Output: CC

Expected output: CC

$SC_{88}$: ${\mathrm{Input}}_{1}$: user enters train or flight number and leaving date → $SyRp_{88.1}$: system saves the information

$ESyRp_{88.1}$: system saves the information

Output: CC

Expected output: CC

## 4. Results

**Figure 6:**
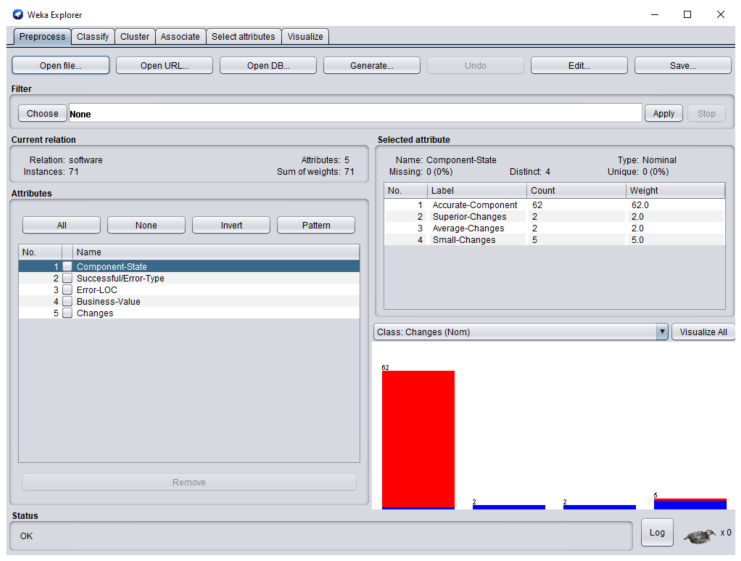
Component-state attribute of the dataset in Waikato Environment for Knowledge Analysis (WEKA).

**Figure 7:**
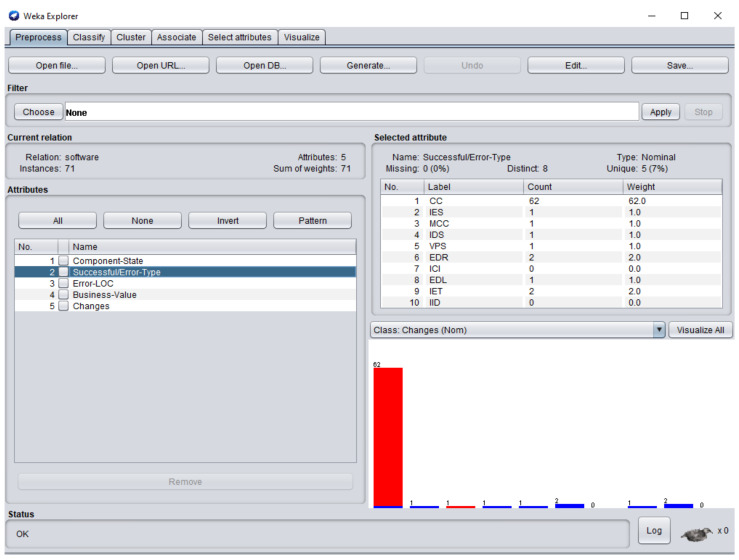
Successful/error-type attribute of the dataset in WEKA.

**Figure 8:**
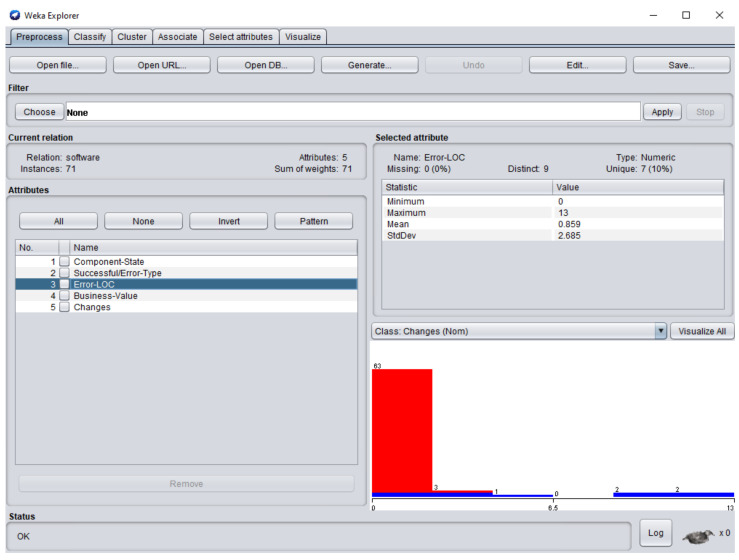
Error-LOC (line of code) attribute of the dataset in WEKA.

**Figure 9:**
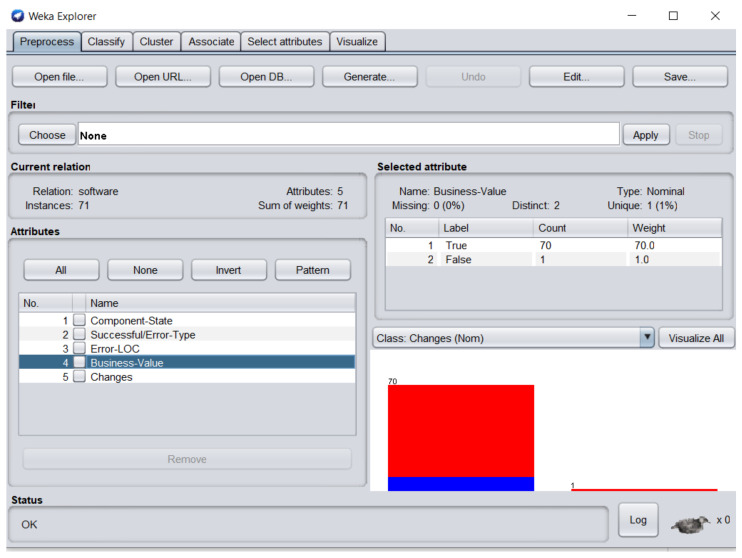
Business-value attribute of the dataset in WEKA.

**Figure 10:**
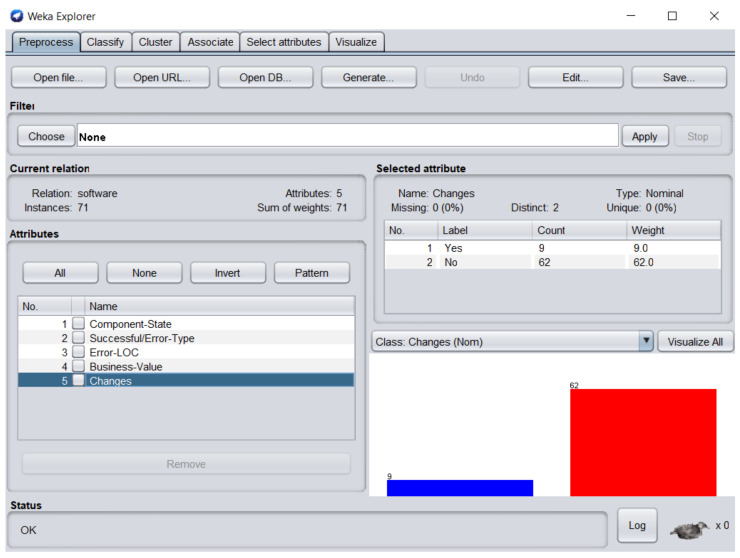
Changes attribute of the dataset in WEKA.

The dataset consisted of 71 instances and 5 attributes: component-state, successful/error-type, error-LOC, business-value, changes. WEKA stands for “Waikato Environment for Knowledge Analysis”, it is a machine learning software introduced by the University of Waikato, New Zealand. WEKA consists of algorithms and visualization tools which are used for predictive modeling and data analysis [37]. Figure 6, Figure 7, Figure 8, Figure 9 and Figure 10 represent the attributes of the dataset used in the WEKA software.

Figure 6 represents component-state attribute; 62 software components were accurate-component (CC), software component $SC_{5}$ and $SC_{17}$ required superior changes, software component $SC_{4}$ and $SC_{49}$ required average changes, whereas small changes were performed in the software components $SC_{6}$, $SC_{26}$, $SC_{27}$ $SC_{51}$, $SC_{60}$. Figure 7 represents successful/error-type attribute. In software components $SC_{4}$, $SC_{5}$, $SC_{6}$ $SC_{6}$, $SC_{26}$, $SC_{17}$, $SC_{26}$, $SC_{27}$, $SC_{49}$, $SC_{51}$, and $SC_{60}$, the error types IDS, IET, MCC, EDR, IET, EDL, IES, VPS, and EDR were extracted respectively. Figure 8 represents the error-LOC attribute. Software components $SC_{4}$, $SC_{5}$, $SC_{6}$ $SC_{6}$, $SC_{26}$, $SC_{17}$, $SC_{26}$, $SC_{27}$, $SC_{49}$, $SC_{51}$, and $SC_{60}$ consisted of 9, 12, 3, 13, 3, 4, 10, 5, and 2 error-LOC respectively.

Figure 9 represents business-value attribute, in which one software component business value was zero. Figure 10 represents changes attribute; 9 software components required changes and 62 software components were perfect.

**Figure 11:**
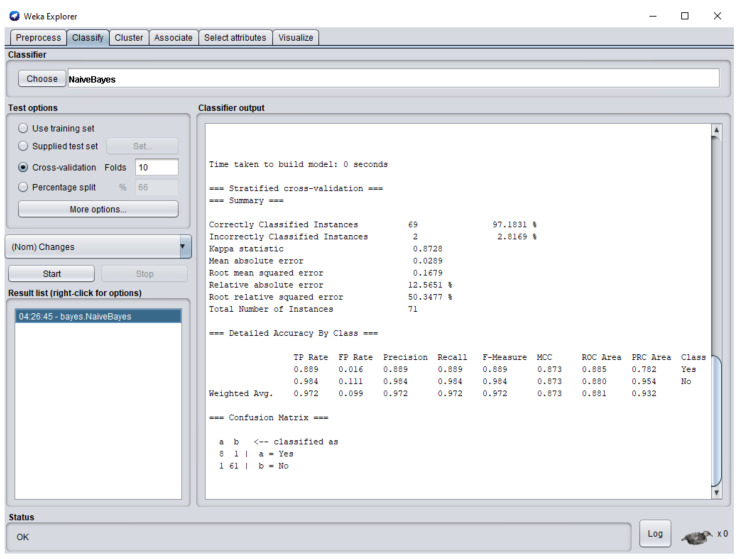
Naïve Bayes classifier results for the software.

Figure 11 represents the Naïve Bayes classifier results, in which 97.18% are correctly classified instances whereas 2.82% are incorrectly classified instances. Reverse engineering is applied when percentage of accuracy is high whereas forward engineering is applied when percentage of accuracy is below 50% and changes required in the software are difficult to handle due to change in business processes or due to advancements in technology. Based on the case study, correctly predicted errors: IES = 1, MCC = 1, IDS = 1, VPS = 1, EDR = 2, ICI = 0, EDL = 1, IET = 2, IID = 0. By substituting the values of errors in Equation (2).

(2) $$Correctly\;Predicted\;Errors\;{\left(CPE\right)}_{\left[Machine\;Learning\right]}=\overset{n}{\underset{j=1}{\sum }}SC_{E_{T}}\;=\overset{n}{\underset{j=1}{\sum }}(SC_{\mathrm{IES}}\;+\;SC_{\mathrm{MCC}}+\;SC_{\mathrm{IDS}}+\;SC_{\mathrm{VPS}}+\;SC_{\mathrm{EDR}}+\;SC_{\mathrm{ICI}}+\;SC_{\mathrm{EDL}}+\;SC_{\mathrm{IET}}+\;SC_{\mathrm{IID}})\;\;=\overset{n}{\underset{j=1}{\sum }}\left(1+1+1+1+2+0+1+2+0\right)=9\;$$

According to Equation (2), total correctly predicted errors are 9, so 9 software components require maintainability, whereas new components (NC) required to be developed according to new functional and nonfunctional requirements are NC = 17. According to the dataset used in the Naïve Bayes classifier, the total number of software components was 71. Software total components (STC) is the sum of previous total components and new components to be developed. The answer is calculated in index value (0–1) whereas 0.29 indicates the maintainability required in the software. By substituting the values of CPE, NC, and STC in Equation (3) given below, software maintainability was determined.

(3) $$Software\;Maintainability_{\left[Machine\;Learning\right]}=\frac{\left(CPE+NC\right)}{STC}\;=\frac{\left(9+17\right)}{88}=0.29$$

In this research, the legacy system was changed according to new requirements of the user. For each new functional and non-functional requirement, new software components were designed, whereas for minor modifications in previous functional and non-functional requirements, the previous software components of legacy system were upgraded. The modification in the system was done by component-based development, in which each software component was validated with a supervised learning approach to detect errors in it. The total number of software components in the complete system is 71. The results generated by the supervised learning approach detected 9 errors in the software components and 62 software components were error-free. Therefore, the correctness rate of the system was 87.32%, whereas the results of Naïve Bayes classifier show that only 2 instances were incorrectly classified, and 69 instances were correctly classified with precision = 0.97.

## 5. Discussion

The results presented in the study have internal and external validity threats to the system. Internal validity: attributes data used in the research significantly depend on age of the legacy system and the number of changes requested in the new software requirements. The change rate in the system increases with time because the systems evolve due to evolutionary changes in the working environment, and faults rate also increases due to new requirements. In order to avoid these threats, we used a requirements validation framework that helps to design the software components according to software requirements. External validity: the threat to validity is the implementation languages, e.g., Visual Basic .Net, C++, Java etc. During the design of the system, completeness and consistency are considered important factors. To avoid this threat, the introduced methodology focused on the correctness of the software components, which reduces the error rate in the software.

## 6. Conclusions

As the number of errors and/or defects increases in the software, the business value of the software also decreases. Reverse engineering can only be applied if there are fewer errors or defects in the software. The business value of the software increases if there is smaller number of errors or defects. Forward engineering is applied in the software having high business value. Illustrating the software components in the form of component-based user interfaces helped in the identification of potential problems in the software. The conducted case study showed that predicted values by WEKA software were approximately equal to the values calculated by the software components. It has been observed that the software components that were developed by considering the inverse requirements were error-free and were easily changeable according to the customer requirements. The design of the software components helped in better understanding of software requirements. The dataset stores complete records about maintainability of the software, as well as about each software component. This approach reduces the fault rate, which has been a challenging task for software engineers.

## Figures and Tables

**Table 1:** Validation of legacy system by supervised learning approach.

$M_{i}SC_{j}$ ^1^	Description		Sf/Et ^2^
$M_{1}:$ Create Account			
$M_{1}SC_{1}$	$R_{1}$ 3: The system shall display text box to enter the first name.$SyRp_{1}$ 4: The system displays a message asking the applicant to enter the first name, only alphabets will be accepted in this text box. This software component will be displayed in the module $M_{3}$, in which first name will be unaltered.	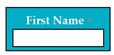	CC
$M_{1}SC_{2}$	$R_{2}:$ The system shall display text box to enter the family name. $SyRp_{2}:$ The system displays a message asking the applicant to enter the family name, only alphabets will be accepted in this text box. Family name will consist of last name and middle name if any. This software component will be displayed in the module $M_{3}$, in which family name will be unaltered.	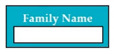	CC
$M_{1}SC_{3}$	$R_{3}:$ The system shall display text box to register with the valid e-mail address. $SyRp_{3}:$ The system displays a message asking the applicant to register with the valid e-mail address (Hotmail/Yahoo/Academic etc.). This software component will be displayed in the module $M_{3}$, in which e-mail address will be unaltered.	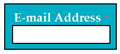	CC
$M_{1}SC_{4}$	$R_{4}:$ The system shall display text box to confirm the e-mail address. $SyRp_{4}:$ The system displays a message asking the applicant to confirm the e-mail address again.	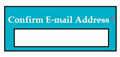	IDS
$M_{1}SC_{5}$	$R_{5}:$ The system shall display text box to enter the date of birth. $SyRp_{5}:$ The system displays a message asking the applicant to enter the date of birth in the format YYYY/MM/DD. If the entered age is above 35 years before the deadline, then the system will display text box in red color. This software component will be displayed in the module $M_{3}$, in which entered date of birth will be unaltered.	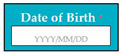	IET
$M_{1}SC_{6}$	$R_{6}:$ The system shall display text box to enter the cell number. $SyRp_{6}:$ The system displays a message asking the applicant to enter the cell number in the format e.g., +86 123-2399-9999, where +86 is country code. The system will send PIN code at the entered cell number.	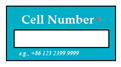	MCC
$M_{1}SC_{7}$	$R_{7}:$ The system shall display text box to enter the PIN. $SyRp_{7}:$ The system displays a message asking the applicant to enter the PIN, that was recently sent by the system at the cell number.	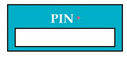	CC
$M_{1}SC_{8}$	$R_{8}:$ The system shall display text box to enter the password. $SyRp_{8}:$ The system displays a message asking the applicant to enter the password. Password must be more than 8 characters, consists of at least 1 uppercase letter (A–Z), lowercase letter (a–z), number (0–9), symbol (&%^$! etc.), whereas space will not be considered.	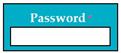	CC
$M_{1}SC_{9}$	$R_{9}:$ The system shall display text box to confirm the password. $SyRp_{9}:$ The system displays a message asking the applicant to confirm the same entered password again.	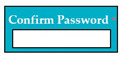	CC
$M_{1}SC_{10}$	$R_{10}:$ The system shall display the submit button. $SyRp_{10}:$ The system displays a message asking the applicant to submit the entered information (from $SC_{1}$ to $SC_{9}$) by using submit button, for creating the new account.	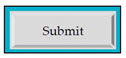	CC
$M_{2}:$ Login			
$M_{2}SC_{11}$	$R_{3}:$ The system shall display text box to enter the E-mail address. $SyRp_{11}:$ The system displays a message asking the applicant to enter the valid e-mail address (Hotmail/Yahoo/Academic etc.) that was used to create the account.	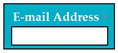	CC
$M_{2}SC_{12}$	$R_{8}:$ The system shall display text box to enter the password. $SyRp_{12}:$ The system displays a message asking the applicant to enter the password. Password must be same that was used for creating the account.	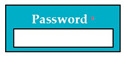	CC
$M_{2}SC_{13}$	$R_{11}:$ The system shall display button to login the system. $SyRp_{13}:$ The system displays a message asking the applicant to login the system. By clicking the login button applicant will have access to the admission application.	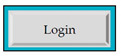	CC
$M_{3}:$ Information			
$M_{3}SC_{14}$	$R_{12}:$ The system shall display text box with entered first name.$SyRp_{14}:$ The system displays text box already containing the first name in it, that was entered in it during the create account module. The blue (lighter 60%) colored text box containing first name indicates that text is unaltered.	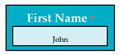	CC
$M_{3}SC_{15}$	$R_{13}$: The system shall display text box with entered family name.$SyRp_{15}:$ The system displays text box already containing the family name in it, that was entered in it during the create account module. The blue (lighter 60%) colored text box containing family name indicates that text is unaltered.	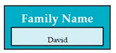	CC
$M_{3}SC_{16}$	$R_{14}:$ The system shall display radio button for the selection of gender. $SyRp_{16}:$ The system displays two radio buttons asking the applicant to select his/her gender (male/female).	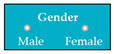	CC
$M_{3}SC_{17}$	$R_{15}:$ The system shall display text box to enter the passport number, issue and expiry date of the passport. $SyRp_{17}:$ The system displays a message asking the applicant to enter the passport number, issue and expiry date of the passport. If the passport expiry date is less than 6 months before the deadline, then the system will display text box in red color.	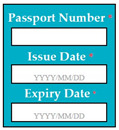	EDR
$M_{3}SC_{18}$	$R_{16}:$ The system shall display text box with entered date of birth. $SyRp_{18}:$ The system displays text box already containing the date of birth in it that was entered in it during the create account module. The blue (lighter 60%) colored text box containing date of birth indicates that text is unaltered.	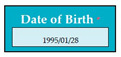	CC
$M_{3}SC_{19}$	$R_{17}:$ The system shall display text box with entered e-mail address. $SyRp_{19}:$ The system displays text box already containing the e-mail address in it, that was entered in it during the create account module. The blue (lighter 60%) colored text box containing e-mail address indicates that text is unaltered.	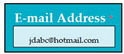	CC
$M_{3}SC_{20}$	$R_{18}:$ The system shall display text box to enter the phone number. $SyRp_{20}:$ The system displays a message asking the applicant to enter phone number in the format e.g., +86-23-999999 where +86 is country code and 23 is area code.	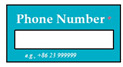	CC
$M_{3}SC_{21}$	$R_{19}:$ The system shall display drop down list for the selection of nationality.$SyRp_{21}:$ The system displays a message asking the applicant to select his/her nationality from the drop-down list.	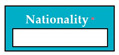	CC
$M_{3}SC_{22}$	$R_{20}:$ The system shall display text box to enter the street address.$SyRp_{22}:$ The system displays a message asking the applicant to enter his/her street address. In the text box alphanumeric data can be entered.	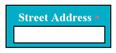	CC
$M_{3}SC_{23}$	$R_{21}:$ The system shall display text box to enter the city name.$SyRp_{23}:$ The system displays a message asking the applicant to enter the city name.	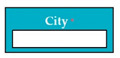	CC
$M_{3}SC_{24}$	$R_{22}:$ The system shall display text box to enter the postal code of city.$SyRp_{24}:$ The system displays a message asking the applicant to enter the postal code of city.	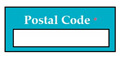	CC
$M_{3}SC_{25}$	$R_{23}:$ The system shall display text box to enter the province.$SyRp_{25}$: The system displays a message asking the applicant to enter the province.	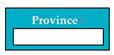	CC
$M_{3}SC_{26}$	$R_{24}:$ The system shall display text box to enter the validity date of address.$SyRp_{26}:$ The system displays a message asking the applicant to enter the validity date of address in the format YYYY/MM/DD.	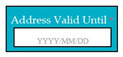	IET
$M_{4}:$ Research Interests			
$M_{4}SC_{27}$	$R_{25}:$ The system shall display drop-down list for the selection of research interests. $SyRp_{27}:$ The system displays a message asking the applicant to select his/her research interests from the drop-down list. The drop-down list will contain radio buttons from which applicant can select more than one his/her research interests.	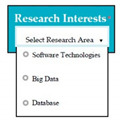	EDL
$M_{4}SC_{28}$	$R_{26}:$ The system shall display button to upload the documents.$SyRp_{28}:$ The system displays a message asking the applicant to upload the documents e.g., CV, awards etc. by clicking the upload button. Following types of documents can be uploaded .doc, .txt, .xls, .rtf, .docx, .jpg, .pdf etc. File size must not be more than 2048 KB, uploaded document must not be password protected.	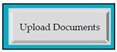	CC
$M_{5}:$ Education			
$M_{5}SC_{29}$	$R_{27}:$ The system shall display text box to enter the name of university last attended.$SyRp_{29}:$ The system displays a message asking the applicant to enter the name of university last attended for degree 1. University name for degree 1 must be entered in reverse sequential order.	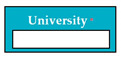	CC
$M_{5}SC_{30}$	$R_{28}:$ The system shall display text box to enter the city name of the university.$SyRp_{30}:$ The system displays a message asking the applicant to enter the city name of the university.	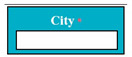	CC
$M_{5}SC_{31}$	$R_{29}:$ The system shall display text box to enter the university website address. $SyRp_{31}:$ The system displays a message asking the applicant to insert the university website address in the text box.	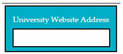	CC
$M_{5}SC_{32}$	$R_{30}:\;$The system shall display drop-down list for the selection of major.$SyRp_{32}:$ The system displays a message asking the applicant to select the major of previous degree from the drop-down list.	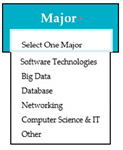	CC
$M_{5}SC_{33}$	$R_{31}:$ The system shall display text boxes to enter the dates of attending the university and approved number of years of the degree. $SyRp_{33}:$ The system displays a message asking the applicant to enter the dates of attending the university, first text box to add the start date of degree, second text box to add the end date of degree and third text box to add the approved number of years of the degree.	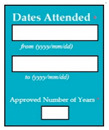	CC
$M_{5}SC_{34}$	$R_{32}:$ The system shall display drop-down list for the selection of title of degree. $SyRp_{34}:$ The system displays a message asking the applicant to select the title of degree from the drop-down list. List consists of Intermediate, Bachelor, Master, PhD, other etc. If the applicant selects the other option from the drop-down list, then text box will appear to write the degree title.	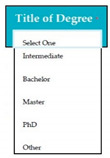	CC
$M_{5}SC_{35}$	$R_{33}:$ The system shall automatically convert Cumulative Grade Point Average (CGPA) according to the university formula. $SyRp_{35}:$ The system displays a message asking the applicant to enter obtained CGPA, maximum and minimum grade point. As 4.0 can be maximum grade point and 2.5 can be minimum grade point adopted by the degree issuing university. CGPA will be automatically converted by the software according to the CGPA formula of admission university. At the back-end formula will be implemented to do the automatic conversion.	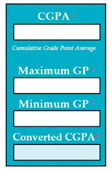	CC
$M_{5}SC_{36}$	$R_{34}:$ The system shall display button to upload the degree. $SyRp_{36}:$ The system displays a message asking the applicant to upload the degree. By clicking the upload button following types of documents can be uploaded .doc, .txt, .xls, .rtf, .docx, .jpg, .pdf etc.	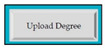	CC
$M_{5}SC_{37}$	$R_{35}:$ The system shall display button to upload the transcript. $SyRp_{37}:$ The system displays a message asking the applicant to upload the transcript. By clicking the upload button following types of documents can be uploaded .doc, .txt, .xls, .rtf, .docx, .jpg, .pdf etc.	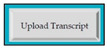	CC
$M_{5}SC_{38}$	$R_{36}:$ The system shall add complete details about another degree, if the applicant clicks on the yes option of the radio button.$SyRp_{38}$: The system displays two radio buttons asking the applicant whether he/she wants to add details about another degree. If the applicant clicks on the yes option of the radio button then the software components from $SC_{29}$ to $SC_{37}$ will be again displayed in the software, so detail about another degree can be added.	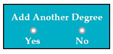	CC
$M_{6}:$ Research Publications			
$M_{6}SC_{39}$	$R_{37}:$ The system shall display drop-down list for the selection of publication category.$SyRp_{39}:$ The system displays a message asking the applicant to select the publication category from the drop-down list.	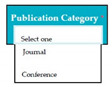	CC
$M_{6}SC_{40}$	$R_{38}:$ The system shall display text box with entered publication category e.g., journal. $SyRp_{40}:\;$The system displays text box already containing the journal as publication category in it that was selected in $SC_{39}$. The blue (lighter 60%) colored text box indicates that text is unaltered.	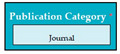	CC
$M_{6}SC_{41}$	$R_{39}:$ The system shall display text box to enter the journal ISSN. $SyRp_{41}:$ The system displays a message asking the applicant to enter the journal ISSN. The system will check whether the research publication is recognized by SCI/SCIE or not.	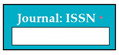	CC
$M_{6}SC_{42}$	$R_{40}:$ The system shall display text box to enter authors of the article published in journal. $SyRp_{42}:$ The system displays a message asking the applicant to enter authors of the article published in journal, in which he/she is author or co-author.	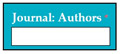	CC
$M_{6}SC_{43}$	$R_{41}:$ The system shall display text box to enter the article title. $SyRp_{43}:$ The system displays a message asking the applicant to enter the article title published in journal.	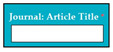	CC
$M_{6}SC_{44}$	$R_{42}:$ The system shall display text box to enter the journal title. $SyRp_{44}:$ The system displays a message asking the applicant to enter the journal title.	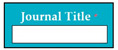	CC
$M_{6}SC_{45}$	$R_{43}:$ The system shall display text box to enter volume number of the journal.$SyRp_{45}:$ The system displays a message asking the applicant to enter volume number of the journal.	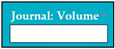	CC
$M_{6}SC_{46}$	$R_{44}:$ The system shall display text box to enter issue number of the journal. $SyRp_{46}:$ The system displays a message asking the applicant to enter issue number of the journal.	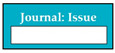	CC
$M_{6}SC_{47}$	$R_{45}:$ The system shall display text box to enter the page numbers of article published in the journal.$SyRp_{47}:$ The system displays a message asking the applicant to enter the page numbers (pp) of article published in the journal.	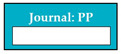	CC
$M_{6}SC_{48}$	$R_{46}:$ The system shall display text box to enter the year in which journal article was published.$SyRp_{48}:$ The system displays a message asking the applicant to enter the year in which journal article was published.	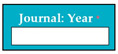	CC
$M_{6}SC_{49}$	$R_{47}:$ The system shall display entered details of the journal article. $SyRp_{49}:$ The system displays text box containing details of the published journal article that were entered in ${\mathrm{SC}}_{41}$ to ${\mathrm{SC}}_{48}$ and also contains buttons to update/delete details of the journal article.	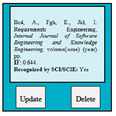	IES
$M_{6}SC_{50}$	$R_{48}:\;$The system shall add complete details about another published article in journal, if the applicant clicks on the yes option of the radio button. $SyRp_{50}:$ The system displays two radio buttons asking the applicant whether he/she wants to add details about another published article in journal. If the applicant clicks on the yes option of the radio button then software components from ${\mathrm{SC}}_{41}$ to ${\mathrm{SC}}_{48}$ will be again displayed in the software, so detail about another published article can be added.	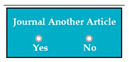	CC
$M_{6}SC_{51}$	$R_{49}:$ The system shall display text box with entered publication category e.g., conference. ${\mathrm{SyRp}}_{51}:$ The system displays text box already containing the conference as publication category in it that was selected in ${\mathrm{SC}}_{39}$. The blue (lighter 60%) colored text box indicates that text is unaltered.	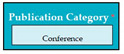	VPS
$M_{6}SC_{52}$	$R_{50}:$ The system shall display text box to enter the conference ISSN. ${\mathrm{SyRp}}_{52}:$ The system displays a message asking the applicant to enter the conference ISSN. The system will check whether the conference is recognized by CCF/IEEE/ACM/Springer. Where CCF stands for China Computer Federation.	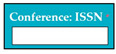	CC
$M_{6}SC_{53}$	$R_{51}:$ The system shall display text box to enter the authors of article published in the conference proceedings. ${\mathrm{SyRp}}_{53}:$ The system displays a message asking the applicant to enter the authors of article published in the conference proceedings, in which he/she is author or co-author.	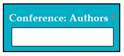	CC
$M_{6}SC_{54}$	$R_{52}:$ The system shall display text box to enter the article title. ${\mathrm{SyRp}}_{54}:$ The system displays a message asking the applicant to enter the article title published in the conference proceedings.	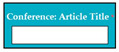	CC
$M_{6}SC_{55}$	$R_{53}:$ The system shall display text box to enter the conference name. ${\mathrm{SyRp}}_{55}:$ The system displays a message asking the applicant to enter the conference name.	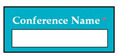	CC
$M_{6}SC_{56}$	$R_{54}:$ The system shall display text box to enter the location of conference. ${\mathrm{SyRp}}_{56}:$ The system displays a message asking the applicant to enter the location of conference (City, Country).	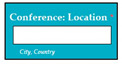	CC
$M_{6}SC_{57}$	$R_{55}:$ The system shall display text box to enter the conference date. ${\mathrm{SyRp}}_{57}:$ The system displays a message asking the applicant to enter the conference date (YYYY/MM/DD).	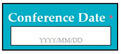	CC
$M_{6}SC_{58}$	$R_{56}:$ The system shall display text box to enter the page numbers of conference article.${\mathrm{SyRp}}_{58}:$ The system displays a message asking the applicant to enter the page numbers (pp) of conference article.	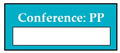	CC
$M_{6}SC_{59}$	$R_{57}:$ The system shall add complete details about another article published in the conference proceedings, if the applicant clicks on the yes option of the radio button.${\mathrm{SyRp}}_{59}:$ The system displays two radio buttons asking the applicant whether he/she wants to add details about another article published in the conference proceedings. If the applicant clicks on the yes option of the radio button then software components from $SC_{52}$ to $SC_{58}$ will be again displayed in the software, so detail about another published article in the conference proceeding can be added.	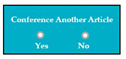	CC
$M_{6}SC_{60}$	$R_{58}:$ The system shall display entered details of the conference article. ${\mathrm{SyRp}}_{60}:$ The system displays text box containing details of the published conference article that were entered in ${\mathrm{SC}}_{52}$ to ${\mathrm{SC}}_{58}$ and also contains buttons to update/delete the conference article.	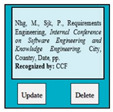	EDR
$M_{7}:$ Job			
$M_{7}SC_{61}$	$R_{59}:$ The system shall display text box to enter the job occupation. ${\mathrm{SyRp}}_{61}:$ The system displays a message asking the applicant to enter the job occupation, if the applicant is employee of any organization.	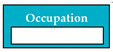	CC
$M_{7}SC_{62}$	$R_{60}:$ The system shall display text boxes to enter the organization address and dates attended the organization. ${\mathrm{SyRp}}_{62}:$ The system displays a message asking the applicant to enter the organization address and dates attended the organization, first text box to add the address of organization, second text box to add start date of job and third text box to add the end date of job or the applicant can enter to-date if his/her job is continuing.	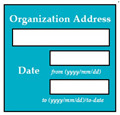	CC
$M_{7}SC_{63}$	$R_{61}:$ The system shall add complete details about another organization, if the applicant clicks on the yes option of the radio button. $SyRp_{63}:$ The system displays two radio buttons asking the applicant whether he/she wants to add details about another organization. If the applicant clicks on the yes option of the radio button then software components ${\mathrm{SC}}_{61}$ and ${\mathrm{SC}}_{62}$ will be again displayed in the software, so detail about organization can be added.	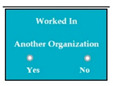	CC
$M_{8}:$ Language			
$M_{8}SC_{64}$	$R_{62}:$ The system shall display drop-down list for the selection of mother tongue. $SyRp_{64}:$ The system displays a message asking the applicant to select mother tongue from the drop-down list of languages.	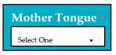	CC
$M_{8}SC_{65}$	$R_{63}:$ The system shall display different levels of written English.$SyRp_{65}:$ The system displays a message asking the applicant to select his/her level of written English. Levels of written English includes excellent, good, fair, none if the applicant cannot write.	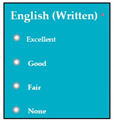	CC
$M_{8}SC_{66}$	$R_{64}:$ The system shall display different levels of spoken English.$SyRp_{66}:$ The system displays a message asking the applicant to select his/her level of spoken English. Levels of spoken English includes excellent, good, fair, none if the applicant cannot speak.	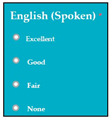	CC
$M_{8}SC_{67}$	$R_{65}:$ The system shall display drop-down list for the selection of language test. $SyRp_{67}:\;$The system displays a message asking the applicant to select the language test in which he/she recently appeared in. List consists of IELTS (Academic), IELTS (General Training), TOEFL (Computer Based), TOEFL (Internet Based), TOEFL (Paper Based).	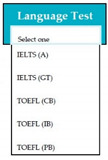	CC
$M_{8}SC_{68}$	$R_{66}$: The system shall display text box to enter the score of language test. $SyRp_{68}:$ The system displays a message asking the applicant to enter the score of language test.	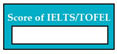	CC
$M_{8}SC_{69}$	$R_{67}:$. The system shall display button to upload the certificate of language test. $SyRp_{69}:$. The system displays a message asking the applicant to upload the certificate of language test. By clicking the upload button following types of documents can be uploaded .doc, .txt, .xls, .rtf, .docx, .jpg, .pdf etc.	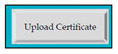	CC
$M_{8}SC_{70}$	$R_{68}:$ The system shall display text boxes to enter the score of International GRE test. $SyRp_{70}:$ The system displays a message asking the applicant to enter the score of International GRE (Graduate Record Examinations) in three portions quantitative, analytical and verbal.	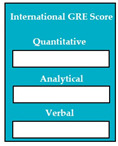	CC
$M_{8}SC_{71}$	$R_{69}:$ The system shall display button to upload the result of GRE test. $SyRp_{71}:$ The system displays a message asking the applicant to upload the result of GRE test. By clicking the upload button following types of documents can be uploaded .doc, .txt, .xls, .rtf, .docx, .jpg, .pdf etc.	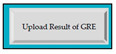	CC
$M_{9}$: Student Record (New Software Components)			
$M_{9}SC_{72}$	$R_{70}:$ The system shall display text box to enter the student ID.$SyRp_{72}:$ The system displays a message asking the applicant to enter the student ID, only alphanumeric data will be accepted in this text box.	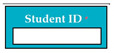	CC
$M_{9}SC_{73}$	$R_{71}:$ The system shall display text box to enter full name.$SyRp_{73}:$ The system displays a message asking the applicant to enter his/her full name.	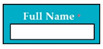	CC
$M_{9}SC_{74}$	$R_{72}:$ The system shall display text box to enter the passport number.$SyRp_{74}$ The system displays a message asking the applicant to enter his/her passport number.	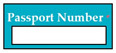	CC
$M_{9}SC_{75}$	$R_{73}$: The system shall display drop-down list for the selection of student category.$SyRp_{75}:$ The system displays a message asking the applicant to select his/her student category from the drop-down list e.g., PhD, Master, Bachelor.	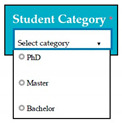	CC
$M_{9}SC_{76}$	$R_{74}:$ The system shall display radio buttons for the selection of address.$SyRp_{76}:$ The system displays a message asking the applicant to select his/her address. If the applicant selects off campus option, then he/she has to enter his/her complete address in the text box.	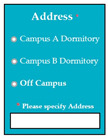	CC
$M_{9}SC_{77}$	$R_{75}:$ The system shall display text boxes to enter emergency details.$SyRp_{77}:$ The system displays a message asking the applicant to enter emergency details e.g., name, contact number.	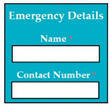	CC
$M_{9}SC_{78}$	$R_{76}:$ The system shall display text boxes to enter first entry date and airport city.$SyRp_{78}:$ The system displays a message asking the applicant to enter first entry date and airport city.	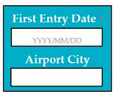	CC
$M_{9}SC_{79}$	$R_{77}:$ The system shall display radio buttons for the selection of COVID-19 test results.$SyRp_{79}:$ The system displays a message asking the applicant to select his/her COVID-19 test results (negative, positive, not tested).	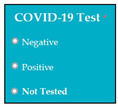	CC
$M_{9}SC_{80}$	$R_{78}:$ The system shall display text boxes to enter visa details.$SyRp_{80}:$ The system displays a message asking the applicant to enter visa details e.g., visa number, visa expiry date.	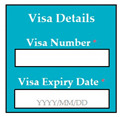	CC
$M_{9}SC_{81}$	$R_{79}:$ The system shall display text boxes to enter details for visa extension letter.$SyRp_{81}:$ The system displays a message asking the applicant to enter details for visa extension letter e.g., student ID, end date of degree.	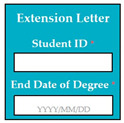	CC
$M_{9}SC_{82}$	$R_{80}:$ The system shall check whether the entered publication is valid for the degree.$SyRp_{82}:$ The system displays a message asking the applicant to enter ISSN number or journal name or title of conference and select indexing attributes (e.g., web of science, SCI, CCF, EI).	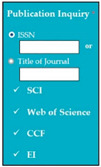	CC
$M_{9}SC_{83}$	$R_{81}:$ The system shall check whether the student is eligible for his/her defense based on his/her published articles.$SyRp_{83}:$ The system displays a message asking the student to enter his/her number of published articles in numeric value and displays message “Candidate is eligible” or “Candidate is not eligible”.	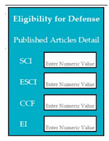	CC
$M_{9}SC_{84}$	$R_{82}:$ The system shall display text box to enter the final defense date.$SyRp_{84}:$ The system displays a message asking the applicant to enter the final defense date, in the format of YYYY/MM/DD.	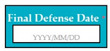	CC
$M_{9}SC_{85}$	$R_{83}:$ The system shall display text boxes to enter student ID and supervisor name.$SyRp_{85}:$ The system displays a message asking the applicant to enter student ID and supervisor name, the system shall display defense information (Student ID, thesis title, date, time, location)	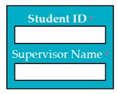	CC
$M_{9}SC_{86}$	$R_{84}:$ The system shall display text boxes to enter student ID and school name.$SyRp_{86}:$ The system displays a message asking the applicant to enter student ID and school name, the system generates transcript.	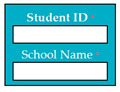	CC
$M_{9}SC_{87}$	$R_{85}:$ The system shall display text boxes to enter student ID and degree completion date.$SyRp_{87}:$ The system displays a message asking the applicant to enter student ID and degree completion date, the system generates university leaving clearance form for signatures.	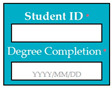	CC
$M_{9}SC_{88}$	$R_{86}:$ The system shall display text boxes to enter train number/flight number and leaving date.$SyRp_{88}:\;$The system displays a message asking the applicant to enter train number/flight number and leaving date, the system generates leave letter.	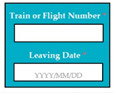	CC

^1^$M_{i}SC_{j}$ stands for $Module_{i}\;Software\;Component_{j}$, (1 ≤ *i* ≤ n), (1 ≤ *j* ≤ n). ^2^ Sf/Et stands for successful and error types respectively, successful denoted by CC (Correct Component), error types: IES (Incomplete Erroneous Specifications), MCC (Misinterpretation of Customer Communication), IDS (Intentional Deviation from Specification), VPS (Violation of Programming Standards), EDR (Error in Data Representation), EDL (Error in Design Logic), ICI (Inconsistent Component Interface), IET (Incomplete or Erroneous Testing). ^3^ $R_{l}$ stands requirements, (1 ≤ *l* ≤ n). ^4^ $SyRp_{m}$ stands for system response, (1 ≤ *m* ≤ n).

## Data Availability

The data used to support the findings of this study are included within the article.
